# Cloning and expression of lipase gene from *Ralstonia eutropha *in *Escherichia coli*

**DOI:** 10.1186/1753-6561-8-S4-P239

**Published:** 2014-10-01

**Authors:** Isabela Cavalcante Rodrigues, Edson Júnior Carmo, Spartaco Astolfi Filho

**Affiliations:** 1 Instituto de Ciências Biológicas, Universidade Federal do Amazonas, Manaus, AM, Brazil

## Background

Lipases are enzymes belonging to the class of hydrolases, acting in the aqueous-organic interface demonstrating considerable levels of activity and stability in aqueous environments and non-aqueous [1]. Its biological function is to catalyze the hydrolysis of long chain triglycerides to form fatty acids and glycerol, in addition to performing esterification [2]. They have wide industrial applicability and can be used in the composition of detergents, in food industry, in wastewater treatment and fine chemistry [3]. The goal this work was clone and express lipase gene from *Rasltonia eutropha *in *Escherichia coli *BL21DE3.

## Methods

The lipase gene was isolated by polymerase chain reaction from a metagenomic library of Terra Preta de Indio of Amazon soil previously constructed in our laboratory. The Lip5 fosmid containing lipase gene *Rasltonia eutropha *was used as template and PCR was performed using Lip5 primers forward and reverse. The amplicons were inserted into cloning vector pCR4 TOPO, multiplied in *E. coli*. The fragment was released by cleavage with restriction enzyme *Eco*RI / *Not*I and subcloned into intracellular expression pGSM vector. *E. coli *BL21DE3 was transformed with recombinant vector and positive transformants were selected by halos formation around colonies in LB solid medium containing 2% Olive Oil as lipase substrate. Recombinant clones are inoculated in LB liquid medium, the culture was incubated with shaking at 37°C until the OD_600 _reached 0.5, IPTG was then added to a final concentration of 1 mM. Cells were collected 8 h after IPTG addition for protein detection by SDS-PAGE.

## Results

A fragment 1200 pb was amplified of Lip5 fosmid and subcloned into intracellular expression pGSM vector. The recombinant plasmid was transformed into *E. coli *BL21DE3. Expression of the lipase gene was induced by IPTG. Translucent degradations halos were observed in solid medium indicating the production of lipase. SDS-PAGE analysis revealed the presence of protein in the supernatant obtained after sonication treatment. The protein had an approximate molecular weight of 39 kDa. We are performing the purification and characterization of recombinant lipase expressed in bacteria and proceeding the cloning in *Pichia pastoris *yeast.
